# The impact of physical exercise on neuroinflammation mechanism in Alzheimer’s disease

**DOI:** 10.3389/fnagi.2024.1444716

**Published:** 2024-08-21

**Authors:** Junhui Hu, Baiqing Huang, Kang Chen

**Affiliations:** ^1^School of Physical Education, West Anhui University, Lu’an, China; ^2^School of Physical Education, Yunnan Minzu University, Kunming, China; ^3^Tianjin Key Laboratory of Exercise Physiology and Sports Medicine, Tianjin University of Sport, Tianjin, China

**Keywords:** Alzheimer’s disease, physical exercise, neuroinflammation, microglial activation, blood-brain barrier integrity

## Abstract

**Introduction:**

Alzheimer’s disease (AD), a major cause of dementia globally, imposes significant societal and personal costs. This review explores the efficacy of physical exercise as a non-pharmacological intervention to mitigate the impacts of AD.

**Methods:**

This review draws on recent studies that investigate the effects of physical exercise on neuroinflammation and neuronal enhancement in individuals with AD.

**Results:**

Consistent physical exercise alters neuroinflammatory pathways, enhances cognitive functions, and bolsters brain health among AD patients. It favorably influences the activation states of microglia and astrocytes, fortifies the integrity of the blood-brain barrier, and attenuates gut inflammation associated with AD. These changes are associated with substantial improvements in cognitive performance and brain health indicators.

**Discussion:**

The findings underscore the potential of integrating physical exercise into comprehensive AD management strategies. Emphasizing the necessity for further research, this review advocates for the refinement of exercise regimens to maximize their enduring benefits in decelerating the progression of AD.

## 1 Introduction

Alzheimer’s disease (AD), the predominant form of dementia, affects millions worldwide, placing significant burdens on healthcare systems and societies (Graff-Radford et al., 2021; Scheltens et al., 2021; Jucker and Walker, 2023). It is characterized by a progressive cognitive decline, disrupting daily activities and leading to loss of independence. Currently, more than 50 million people globally suffer from AD, with projections suggesting that this number could triple by 2050 (Rajan et al., 2021; Hammers et al., 2023). The economic impact of dementia is substantial, with associated costs expected to increase from USD 1 trillion in 2020 to over USD 2 trillion by 2030, exacerbating the social and financial strains on families and economies (Wong, 2020; Alzheimers Dement, 2023; Hammers et al., 2023; Sun J. et al., 2022; Wang Y. et al., 2023). Emerging evidence indicates that neuroinflammation plays a crucial role in the complex pathology of AD, linking molecular abnormalities to clinical symptoms and offering a target for therapeutic interventions (Dias-Carvalho et al., 2024; Veitch et al., 2024). A wide array of traditional Chinese medicines (TCM), such as Ginkgo biloba and Gastrodin, are extensively applied in managing neurodegenerative disorders (Zhou, 2019). These compounds are not only pivotal for neuroprotection, nutritional supplementation, and antioxidation but also show substantial efficacy in mitigating inflammatory responses, a critical aspect in treating AD. Therefore, targeting neuroinflammation not only serves as a potent mechanism to alleviate AD symptoms but also stands as an essential strategy for its prevention and treatment.

In the landscape of AD pathology, neuroinflammation serves as a key mediator of neurodegenerative processes (Ma et al., 2023), driven by the brain’s innate immune cells—microglia and astrocytes (Gao et al., 2023; Langworth-Green et al., 2023; Nakano-Kobayashi et al., 2023; Sangineto et al., 2023; Sun et al., 2023). These cells emit pro-inflammatory cytokines that intensify the accumulation of amyloid-beta plaques and tau tangles, the hallmark features of AD (Bello-Corral et al., 2023; Carna et al., 2023; Sangineto et al., 2023). This inflammatory cycle not only accelerates neuronal damage but also impairs the blood-brain barrier, further driving neurodegeneration. Amid these challenges, physical exercise emerges as a promising non-pharmacological intervention (Lepiarz-Raba et al., 2023; Amar et al., 2024; Dias-Carvalho et al., 2024; Keiser et al., 2024; MoTrPAC Study Group, Lead Analysts and MoTrPAC Study Group, 2024). According to Ayari et al. (2023) demonstrate, physical exercise effectively mitigates these inflammatory responses, shifting cellular phenotypes toward anti-inflammatory states, thus enhancing neuroprotection and cognitive resilience.

Our comprehensive review elucidates the complex interactions between physical exercise and neuroinflammation in AD. Integrating findings from molecular biology, neuroimmunology, and clinical studies, we establish compelling evidence that regular physical exercise ameliorates the inflammatory processes fundamental to AD pathology. Our analysis underscores exercise’s critical role in AD management strategies, demonstrating that physical exercise not only modifies the disease’s trajectory but also significantly enhances quality of life for affected individuals. Highlighting a gap in current research, our review advocates for systematic studies to fine-tune exercise protocols and to explore their sustained effects, opening new avenues in both preventive and therapeutic strategies against this incapacitating condition.

## 2 Characteristics of AD

**FIGURE 1:**
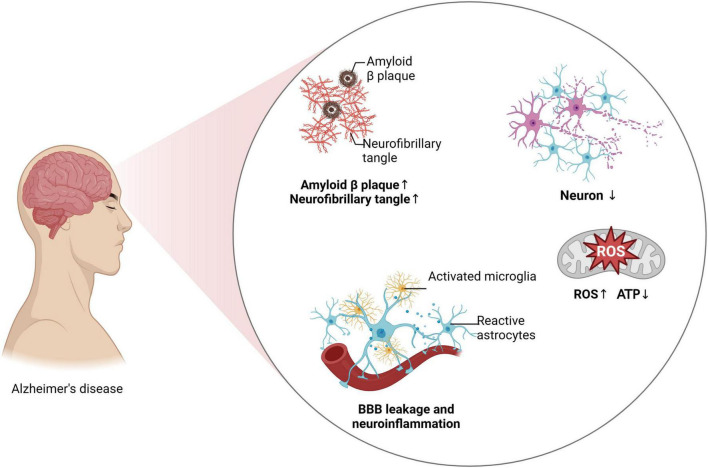
Neuroinflammation and AD progression.

AD represents a principal cause of dementia among the elderly, marked by progressive memory loss, executive dysfunction, and diminished cognitive abilities across various domains (Mathys et al., 2023; Millet et al., 2024). The amyloid hypothesis, proposed in 1984 by Glenner et al. (1984), catalyzed extensive research into AD pathogenesis, introducing a focus on amyloid-beta (Aβ) accumulation (Glenner et al., 1984). Subsequent theories, including the tau hypothesis (Wood and Zinsmeister, 1989), the mitochondrial cascade hypothesis, and the immune dysfunction hypothesis, have broadened our comprehension of the multifactorial nature of AD (Arshavsky, 2020; Ashleigh et al., 2023; Chatanaka et al., 2023; Swerdlow, 2023). Despite advances, the pathogenesis of AD remains elusive, with no single theory fully elucidating its mechanisms, often leading to the failure of therapeutic strategies that target isolated pathways (Boxer and Sperling, 2023). Research consistently associates AD pathogenesis with the abnormal buildup of Aβ and tau protein hyperphosphorylation, both contributing to neuronal degeneration and loss of synaptic plasticity, progressively impairing cognitive functions (Boxer and Sperling, 2023; Chen and Yu, 2023; Kim et al., 2023; Yadollahikhales and Rojas, 2023; Jia et al., 2024; Litvinchuk et al., 2024). Aβ, a cleavage product of the amyloid precursor protein (APP) by β- and γ-secretase, particularly forms Aβ42, a peptide prone to aggregation. Over time, these peptides polymerize into protofibrils, accumulate as amyloid plaques, disrupt cellular communication, activate glial cells, and trigger inflammatory responses, culminating in neuronal death and brain tissue damage (Caraci et al., 2023; Plascencia-Villa and Perry, 2023). In a healthy state, tau protein stabilizes microtubules and supports intraneuronal transport. However, in AD, tau becomes hyperphosphorylated, detaches from microtubules, and forms neurofibrillary tangles (NFTs) within neurons, disrupting transport and leading to neuronal death (Dutta et al., 2023; Ferrer, 2023). The mitochondrial cascade hypothesis posits that mitochondrial dysfunction early in AD escalates reactive oxygen species production and diminishes energy supply, exacerbating the brain’s inflammatory response (de Veij Mestdagh et al., 2023). This inflammatory environment further promotes Aβ deposition and tau phosphorylation, exacerbating mitochondrial dysfunction and precipitating a progressive decline in cognitive abilities in AD patients. These hypotheses collectively underscore the critical role of neuroinflammation in AD pathology and highlight the imperative for in-depth research into neuroinflammatory mechanisms to develop effective therapeutic strategies (Figure 1).

## 3 Neuroinflammation and AD

**FIGURE 2:**
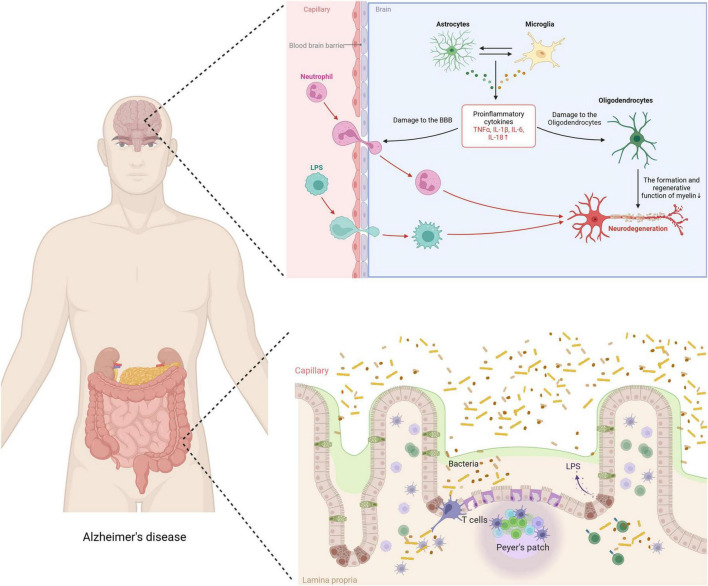
Impact of exercise on neuroinflammation mechanism in AD.

Recent research emphasizes the crucial role of neuroinflammation in AD pathogenesis, particularly highlighting the intricate mechanisms of tau protein modification and systemic inflammation (Chen and Yu, 2023). Excessive phosphorylation of tau protein, a defining characteristic of AD, disrupts microtubule stability, impairs neuronal function, and leads to cell death. Additionally, various post-translational modifications exacerbate this condition by enhancing tau aggregation and neurofibrillary tangle formation, significantly impairing cognitive functions (Figure 2). Moreover, central nervous system (CNS) immune cells, such as microglia, astrocytes, and oligodendrocytes, shift from a state of homeostasis to a pro-inflammatory state in response to AD-associated stimuli, further accelerating disease progression (Singh, 2022). Beyond central mechanisms, recent studies have elucidated the significant contributions of peripheral immune cells, including T cells, in exacerbating neuroinflammation and cognitive decline in AD. These peripheral immune cells not only intensify neuroinflammatory responses but also traverse the compromised blood-brain barrier (BBB), interacting with CNS resident cells to promote inflammatory pathways and cognitive deterioration (Xu and Jia, 2021; Berriat et al., 2023; Jorfi et al., 2023; Zhang et al., 2024). Additionally, changes in the gut microbiome and its metabolic products not only affect brain health but may also facilitate AD progression, illustrating the link between peripheral inflammation and central pathology (Mou et al., 2022). This body of evidence suggests that aging and environmental factors compromise both CNS and peripheral circulatory system functions, fostering a persistent inflammatory response that, in turn, impairs cognitive function.

### 3.1 Inflammation in microglia

Microglia, the brain’s resident immune cells, are pivotal in maintaining neuronal health, responding to injury or disease, and modulating brain development and function (Schafer et al., 2023). They react to pathological changes such as the deposition of Aβ and tau proteins by adopting various activation states, influenced by the local microenvironment. This complexity surpasses the traditional M1/M2 classification, prompting recent studies to advocate a spectrum model of microglial activation that reflects their diverse roles in neuroinflammation and neurodegenerative diseases (Paolicelli et al., 2022). These activation states are dynamic, adjusting to changes such as cytokine levels, damaged neurons, and infections, with functions ranging from promoting inflammation to aiding tissue repair and clearing debris.

In early AD, microglia strive to curb the spread of pathology by phagocytizing Aβ and releasing anti-inflammatory factors (Johnson et al., 2020). However, as AD progresses, the persistent accumulation of Aβ and tau proteins shifts microglial activation from a tissue-repairing to a pro-inflammatory state, potentially hastening neurodegeneration. This transition is marked by increased release of pro-inflammatory cytokines, including tumor necrosis factor-alpha, interleukin-1 beta (IL-1β), and interleukin-6 (IL-6), which not only exacerbate Aβ production but also hinder its clearance (Sun J. et al., 2022; Miao et al., 2023). Additionally, activation of the NLRP3 inflammasome in microglia promotes IL-1β release, closely linked to Aβ aggregation and pathological tau phosphorylation (Zhang Y. et al., 2021). These findings underscore the dynamic and responsive roles of microglia in AD, continuously adapting to their microenvironment through various activation states.

### 3.2 Inflammation in astrocytes

Astrocytes, the most numerous glial cells in the central nervous system, are essential in both normal brain physiology and pathological conditions (Giovannoni and Quintana, 2020). In AD, factors like cytokines, tissue conditions, and interactions with other cell types can prompt changes in astrocyte activation states, influencing disease progression. Recent research emphasizes the dynamic nature of astrocyte activation, demonstrating that these cells exhibit a wide range of functional states beyond simple harmful or beneficial categorizations (Escartin et al., 2021). This variability stems from complex and evolving interactions within the neural microenvironment, highlighting the multifaceted roles of astrocytes in both neural physiology and pathology.

In AD, astrocytes perform a dual role, both protecting against and potentially exacerbating the disease (Jiwaji et al., 2022; Ju et al., 2022). As AD advances, astrocytes respond to brain injury signals, such as the accumulation of Aβ and neuronal damage, by altering their phenotypic expression (Uddin and Lim, 2022). These changes involve a complex array of cytokines and signaling pathways, enabling astrocytes to both promote and inhibit inflammatory responses. Notably, astrocytes primarily detect aggregated Aβ through toll-like receptors (TLRs), especially TLR4 (Gambuzza et al., 2014; Dabi et al., 2023), and receptor for advanced glycation end products (RAGE) (Zhang et al., 2018; He et al., 2022). This interaction activates the nuclear factor kappa B (NF-κB) signaling pathway, leading to the production of inflammatory factors such as tumor necrosis factor-alpha (TNF-α) and IL-1β (Clark et al., 2021). These cytokines further stimulate surrounding microglia and other immune cells, intensifying local inflammatory responses. Additionally, the neuroprotective roles of astrocytes should not be underestimated, as they can secrete neurotrophic factors like brain-derived neurotrophic factor (BDNF) and neurotrophin-3 (NT-3), supporting neuronal survival and enhancing synaptic plasticity (Kahn et al., 1999; Wrann et al., 2013; Khorooshi et al., 2021). However, in later stages of AD, prolonged exposure to chronic inflammation and high Aβ levels may lead astrocytes to an overly reactive state. This “reactive astrocyte” condition is characterized by the release of neurotoxic substances such as glutamate and pro-inflammatory mediators, further deteriorating the brain’s pathological environment (Escartin et al., 2021). In summary, the role of astrocytes in AD is complex and influenced significantly by their capacity to adapt to the evolving pathological landscape. Targeting the diverse functions of astrocytes may offer new avenues for therapeutic interventions aimed at modulating neuroinflammation and slowing AD progression.

### 3.3 Inflammation in oligodendrocytes

Oligodendrocytes, the myelin-forming cells of the CNS, not only play roles in myelination and repair but also have significant interactions with CNS immune cells, such as microglia (Kuhn et al., 2019). These cells produce extensive amounts of cell membrane during their differentiation process to form myelin, which is critical for tightly wrapping around axons of neurons (Kuhn et al., 2019). This myelination significantly enhances and speeds up the conduction of neural impulses, a vital method of intercellular communication. In the neuroinflammatory environment of AD, the behavior of oligodendrocyte precursor cells (OPCs) is affected, which in turn impacts their differentiation and myelin regeneration capabilities (Sadick et al., 2022). The inflammatory environment indirectly affects the function and maturation of OPCs by influencing growth factors and cellular signaling pathways, such as PI3K and p38 MAPK (Huang et al., 2024). Interestingly, the interactions between oligodendrocytes and microglia can influence myelin formation and regeneration. Research has shown that microglia can have a detrimental effect on oligodendrocytes under inflammatory conditions (McNamara et al., 2023). However, under normal conditions, their interaction, through the release of factors that promote myelination, supports myelin recovery. Furthermore, the communication between oligodendrocytes and astrocytes is also linked to the autophagy processes of neurons. In AD, impaired autophagy may exacerbate neuroinflammation and neurodegenerative processes, affecting the communication functions of astrocytes and oligodendrocytes, subsequently impacting the overall function of the CNS (Sadick et al., 2022). In summary, the interactions between oligodendrocytes and the immune system in AD occur through complex cellular and molecular mechanisms involving microglia, astrocytes, and other immune cells. These interactions not only influence the progression of the disease but may also provide potential targets for developing new therapeutic strategies.

### 3.4 Gut inflammation

The link between gut inflammation and AD has garnered growing scientific interest. Recent studies indicate that gut microbiota dysbiosis significantly affects brain function and pathology through various mechanisms, critically influencing AD’s pathogenesis (Chen C. et al., 2022; Ferreiro et al., 2023; Xia et al., 2023; Zhang et al., 2023; Chen et al., 2024). Supported by recent research, the gut-brain axis hypothesis suggests that changes in gut microbiota impact brain function and health through several pathways (Das and Ganesh, 2023). Gut inflammation, commonly triggered by dysbiosis, activates gut-associated lymphoid tissues (GALT), crucial for immune modulation (Saksida et al., 2018). This activation stimulates immune cell proliferation, especially T cells, leading to the production of various cytokines and chemokines (Saksida et al., 2018). These immune mediators increase inflammatory cytokine production, which can breach the BBB and intensify neuroinflammatory responses in the brain—processes closely related to AD pathology (Huang et al., 2021). Emerging evidence highlights the vital roles of CNS glial cells and peripheral immune cells, especially T cells, in exacerbating neuroinflammation and cognitive decline in AD. Once activated, these T cells can cross the BBB, whose permeability increases due to systemic inflammation (Li et al., 2021, Lee et al., 2023). Within the brain, they interact with microglia and other immune cells, intensifying the inflammatory environment. This not only worsens the production of AD-related pathological markers, like amyloid-beta plaques and neurofibrillary tangles, but also contributes to neuronal damage and cognitive decline (Minter et al., 2016; Mila-Aloma et al., 2020). Studies by Cattaneo et al. (2017) show that inflammatory cytokines, such as IL-6 and TNF-α, often produced by activated T cells, are significantly elevated in AD patients’ brains and positively correlate with disease severity. Additionally, gut microbiota indirectly modulates brain function by influencing metabolic pathways. Notably, it produces short-chain fatty acids (SCFAs), which are anti-inflammatory mediators that enhance BBB function and provide neuroprotective effects (Parada Venegas et al., 2019). SCFAs also regulate T-cell function, influencing systemic inflammation and potentially affecting the immune pathways involved in AD (Parada Venegas et al., 2019; Ratajczak et al., 2019). Therefore, a reduction in beneficial metabolic products due to changes in gut microbiota may increase the risk of AD. Additionally, dysbiosis could influence brain health and pathological states by affecting communication pathways within the gut-brain axis, such as the vagus nerve. This nerve is crucial in linking gut inflammation to AD due to its regulatory roles in inflammatory responses and neurotransmission.

## 4 Benefits of physical exercise for AD

The development of pharmacological interventions for AD has spanned decades, yet the majority of these efforts have not been successful (Stoiljkovic et al., 2021; Majidazar et al., 2022; Sperling et al., 2023; van Dyck et al., 2023). Currently, common non-pharmacological interventions include cognitive training, lifestyle modifications such as adherence to a Mediterranean diet, physical exercise, and adequate sleep, as well as innovative approaches like music therapy and light stimulation (Table 1; Quail et al., 2020; Sabbagh et al., 2020; Fortier et al., 2021; Sun Y. et al., 2022; Khemka et al., 2023; Nissim et al., 2023). Among these, physical exercise offers multifaceted benefits for AD management. It not only enhances cardiorespiratory health but also improves circulatory functions, thereby increasing cerebral blood flow. Moreover, physical exercise stimulates the release of various neurotrophic factors, such as BDNF and glial cell line-derived neurotrophic factor (GDNF). Additionally, a sedentary lifestyle is recognized as one of the risk factors for AD. Research indicates that approximately 35% of AD cases can be attributed to nine modifiable risk factors, with physical inactivity being a significant contributor (Zhang X. X. et al., 2021; Chen K. et al., 2022). Numerous studies have documented the beneficial effects of physical exercise as a non-invasive intervention on both the prevention and treatment of AD (Table 2; Hoffmann et al., 2016; Cox et al., 2019; Yu et al., 2021). A meta-analysis revealed that regular exercise can reduce the risk of developing AD by 45% (Iso-Markku et al., 2022), with other studies supporting similar findings (Mendez Colmenares et al., 2021; Lopez-Ortiz et al., 2023; Roy et al., 2023). Notably, a key factor in exercise intervention is initiating it in the early pathological stages of the disease. If the intervention occurs in the later stages of AD, the benefits of exercise in counteracting plaque formation and the deterioration of cognitive abilities are significantly reduced (Herring et al., 2016). Additionally, studies in mouse models suggest that voluntary exercise may be more beneficial than forced exercise for better treatment or alleviation of disease progression (Yuede et al., 2009). Emerging neuroimaging evidence further substantiates the neural impacts of physical exercise on AD, elucidating its role in modulating brain structure and function. For example, van der Kleij et al. (2018) observed that moderate-to-high intensity aerobic exercise did not significantly enhance cerebral blood flow (CBF) in AD patients over 16 weeks, suggesting the necessity for longer duration or earlier initiation of exercise interventions. In contrast, aerobic exercises, such as walking and dancing, improve white matter integrity in brain regions critical for cognitive processing, indicating potential for neuroplastic adaptations. Additionally, higher physical activity levels with enhanced white matter integrity, reinforcing the concept that sustained physical activity may serve as a protective strategy against AD progression (Mendez Colmenares et al., 2021).

**TABLE 1:** Overview of non-pharmacological interventions for managing AD.

Intervention method	Main features	References
Cognitive training	Enhances memory, attention, and problem-solving abilities through specifically designed cognitive tasks, stimulating brain activity and enhancing neuroplasticity.	Ali et al., 2022
Mediterranean diet	Rich in vegetables, fruits, whole grains, olive oil, and moderate amounts of fish, it supports cardiovascular health and reduces the risk of AD.	Hoscheidt et al., 2022
Physical exercise	Improves cardiovascular health, increases cerebral blood flow, and promotes the release of neurotrophic factors, improving brain health.	Delgado-Peraza et al., 2023
Adequate sleep	Good sleep helps in clearing toxins from the brain, including beta-amyloid, crucial for maintaining cognitive health.	Zimmerman et al., 2024
Music therapy	Uses elements of music to evoke emotional responses and memories, improving mood and social interaction capabilities, and potentially enhancing cognitive functions.	Reschke-Hernandez et al., 2023
Light stimulation	Employs specific frequency light stimulation to modulate brain activity, aiming to restore normal communication between neurons, especially in AD-related brain areas.	Agger et al., 2023

**TABLE 2:** Effects of exercise on AD outcomes.

Subjects	Intervention groups	Intervention program	Main effects	References
AD patients	Aerobic exercise	12 weeks of treadmill training, 1 h/day, 5 days/week.	Increases synaptic flexibility, memory, number of synapses, and synaptic structure.	Pahlavani, 2023
Elderly, MCI and AD patients	Resistance exercise	Recommended by WHO.	Improves memory, cognitive functions, neurotrophic factors, and reduces Aβ deposition.	Azevedo et al., 2023
AD patients and animal models	Physical exercise	Physical exercise.	Reduces Aβ aggregates, NFTs, inflammatory processes, and improves cognitive functions.	Andrade-Guerrero et al., 2023
AD patients and mice models	Exercise mimetics and physical exercise	Various protocols including treadmill training.	Modulates innate immune responses, reduces neuroinflammation, and attenuates AD progression.	Wang M. et al., 2023; Zhao, 2024
Mice: young, middle-aged, old	Running exercise training	Weekly running based on maximum running speed tests.	Improves cognitive function and reverses hippocampal neuron loss.	Han et al., 2023
Mice models	Chronic unpredictable stress (CUS)-exposed	4 weeks of running exercise.	Alleviated depressive-like behaviors, restored M1/M2 microglial balance, and regulated pro-/anti-inflammatory cytokine production. Effects possibly mediated by adiponectin/AdipoR1 signaling.	Liu et al., 2024
AD patients	Cycle and stretch	6 months of cycling and stretching.	Exercise may reduce decline in global cognition in older adults with mild-to-moderate AD dementia.	Yu et al., 2021
AD patients	High intensity aerobic exercise	60 min sessions three times a week for 16 weeks.	Exercise reduced neuropsychiatric symptoms in patients with mild AD.	Hoffmann et al., 2016
Elderly	Moderate-to-high intensity aerobic exercise	16 weeks physical exercise.	Aerobic exercise improves VO_2_peak in community-dwelling patients with mild AD.	Sobol et al., 2018
AD patients	Moderate-high-intensity aerobic and strength training	72 treatment sessions (90 min each, 3 times per week for 24 weeks).	Exercise training improves peripheral vascular function in AD.	Pedrinolla et al., 2020
AD patients	Moderate-intensity aerobic exercise	16 weeks aerobic stepping exercise.	Improve cognitive functions.	Song and Yu, 2019

Recent extensive research has elucidated the molecular mechanisms by which exercise improves AD. Studies in APP/PS1 mice have shown that both active and forced exercise interventions can effectively reduce brain Aβ and NFT deposits, with improvements in learning and memory capabilities. Kim’s study identified that a 12-week exercise intervention significantly improved brain Aβ deposition and Tau protein phosphorylation (Kim et al., 2019). Additionally, a reduction in hippocampal neuron loss and an increase in new neurons in the Cornu Ammonis 3 (CA3) area and the dentate gyrus were observed (Kim et al., 2019). These results are also supported by other AD model mouse studies (Thomas et al., 2020). Exercise has been found to play a crucial role in the prevention and treatment of AD through muscle-derived myokines, such as BDNF, Irisin, Cathepsin B, clusterin (CLU), and Glycosylphosphatidylinositol-specific phospholipase D1 (GPLD1), which have been extensively reported to improve AD symptoms through various pathways (Ying et al., 2008; Horowitz et al., 2020; de Miguel et al., 2021; Chen K. et al., 2022). BDNF, a growth factor in the neurotrophic factor family, plays crucial roles in regulating axonal growth, synaptic plasticity, and hippocampal neurogenesis. Exercise interventions have been shown to increase the total brain synthesis of BDNF two to three times, with circulating levels of BDNF correlating with brain BDNF levels in AD patients (Erickson et al., 2011). Both acute and chronic exercise significantly increase systemic BDNF levels. Irisin, a muscle factor induced by exercise, has recently attracted widespread attention in the biomedical field. This protein, encoded by the FNDC5 gene and released through exercise, has been shown to cross the blood-brain barrier, affect brain function, and potentially influence the progression of various neurodegenerative diseases (Hecksteden et al., 2013). In AD, Irisin has been found to enhance neuronal survival and reduce toxicity caused by Aβ. Its protective effects may be mediated through the activation of the PI3K/Akt signaling pathway, enhancing cellular resistance to oxidative stress (Chen K. et al., 2022). Cathepsin B, a lysosomal enzyme belonging to the cysteine protease family, plays a significant role in clearing Aβ and regulating inflammation in AD (Chae et al., 2023). CLU and GPLD1, muscle factors discovered in recent years, are closely associated with Aβ metabolism, clearance, and inflammation (Horowitz et al., 2020; de Miguel et al., 2021). Overall, physical exercise has a positive effect on the prevention and treatment of AD, with earlier intervention times yielding better results, primarily due to the action of exercise-induced muscle factors.

## 5 Exercise and neuroinflammation

In AD, immune-associated neuroglial cells such as microglia, astrocytes, and oligodendrocytes exhibit functional abnormalities leading to chronic neuroinflammation, which accelerates the progression of the disease. During the Mild Cognitive Impairment (MCI) stage, the integrity of the BBB diminishes (Montagne et al., 2020), and its permeability increases, enabling peripheral inflammatory activators like lipopolysaccharides (LPS) to penetrate the brain and activate immune-related cells. Research has shown that both the gut barrier and the BBB are compromised in AD patients. The interaction of LPS with Toll-like receptor 4 (TLR4) on immune cells in the gut wall triggers inflammation, increasing intestinal permeability and allowing LPS to enter the bloodstream (Kerr et al., 2022). LPS, a potent immune activator found in the cell walls of Gram-negative bacteria, interacts with TLR4 receptors on neuroglial cells in the brain through the compromised BBB in AD patients, further triggering a cascade of inflammatory responses that form a vicious cycle. Thus, targeting these inflammatory responses could significantly impact the prevention and treatment of AD. Extensive research indicates that physical exercise, as a non-invasive intervention, plays a positive role in preventing and treating AD, particularly in slowing down inflammatory responses (Scheffer and Latini, 2020; Da Rosa et al., 2023; Gothe et al., 2023). The World Health Organization (WHO) recommends that all adults engage in 150–300 min of moderate-intensity physical exercise or 75–150 min of vigorous-intensity physical exercise per week to reduce the risk of AD (Chen K. et al., 2022). The benefits of exercise on inflammation depend largely on its intensity, duration, and individual variability. Studies have shown that regular moderate-intensity exercise can enhance immune system function and improve the body’s inflammatory state by promoting the hypothalamic-pituitary-adrenal axis, improving the cellular survival environment, anti-apoptosis, optimizing autophagy, and regulating endocrine functions. Choi’s study found that four weeks of low-intensity aerobic exercise inhibited the immune-inflammatory response in an LPS-induced inflammation animal model (Choi et al., 2024). In contrast, high-intensity strenuous exercise may damage muscles, activate inflammatory responses, and consume glycogen to release ROS and reactive nitrogen species (RNS), thereby suppressing immune system function and creating a detrimental cycle (Powers et al., 2020). Thus, moderate-intensity exercise is particularly effective at reducing inflammatory responses through immune regulation, enhancing the body’s defense capabilities and potentially preventing neurodegenerative diseases. These results suggest that exercise could be a key method to modulate immune and inflammatory responses.

## 6 Exercise improves AD by alleviating inflammation

**FIGURE 3:**
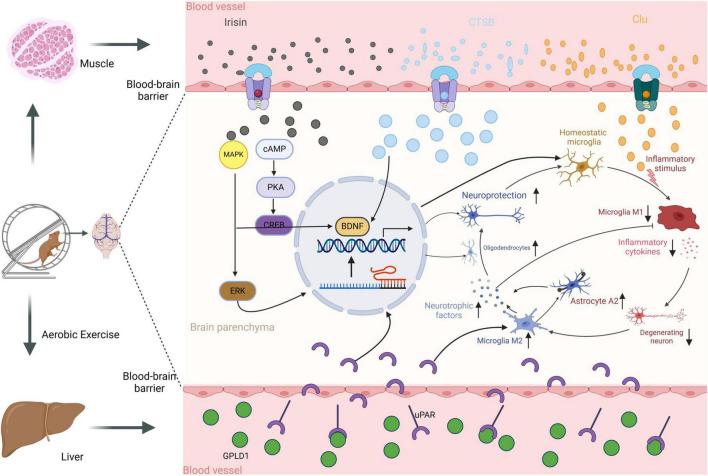
Systemic effects of exercise in modulating AD pathology.

AD is categorized into two types: eFAD and LOAD. The LOAD accounts for over 90% of all cases. Thus, preventing and treating LOAD is particularly crucial. As people age, they accumulate a chronic low-level state of inflammation, which is believed to be a major driver in the development of various chronic diseases during aging. For instance, aging leads to a decline in immune system function, characterized by diminished T-cell functionality, loss of immune memory, and increased activation of inflammatory cells (Mogilenko et al., 2021). These changes reduce the body’s defense against pathogens and increase chronic inflammation. Furthermore, aging cells release various pro-inflammatory cytokines, chemokines, and proteases, a phenomenon known as the senescence-associated secretory phenotype (SASP) (Birch and Gil, 2020). SASP can enhance inflammation in surrounding tissues, further driving the aging process and increasing the risk of metabolic dysfunctions such as obesity and type 2 diabetes, which can induce a systemic state of chronic inflammation. Adipose tissue, especially visceral fat, produces various inflammatory mediators, like TNF-α and IL-6, which can accelerate the aging process (Victorelli et al., 2023). These physiological processes all contribute to a persistent state of chronic inflammation, ultimately exacerbating AD pathology. Interestingly, exercise has been found effective in regulating the body’s inflammatory response. Regular and routine exercise can alleviate chronic inflammation and delay aging by inhibiting pro-inflammatory and enhancing anti-inflammatory factors (Figure 3). In the brain, exercise positively regulates immune-related cells. For microglia, exercise can shift them from a pro-inflammatory M1 state to an anti-inflammatory M2 state, where M2 microglia release various anti-inflammatory factors, such as IL-10 and TGF-β, promoting Aβ clearance and neural repair. This shift reduces the production of pro-inflammatory factors like TNF-α and IL-1β, helping to alleviate AD-related neuroinflammation (Mee-Inta et al., 2019; Han et al., 2023). For astrocytes, physical exercise can reduce their reactivity, moderating their morphology and function to lessen the release of pro-inflammatory mediators. Exercise also enhances the neurotrophic support of astrocytes, including regulating neurotransmitter clearance and providing metabolic support, thereby helping to maintain neuronal health (Liu et al., 2024). For oligodendrocytes, exercise may support survival and function by promoting the release of growth factors such as BDNF and nerve growth factor (NGF), aiding in myelination and repair of neural fibers (Luo et al., 2019). Furthermore, exercise helps enhance BBB structure and function by reducing inflammatory cytokine production and improving endothelial cell function. This includes increasing the expression of tight junction proteins and reducing BBB permeability, thus preventing harmful substances from entering the brain (Lee et al., 2021). For peripheral circulation inflammation, exercise also has a regulatory effect, especially on the inflammatory response of the gut microbiota. Studies have shown that exercise can alter the composition of the gut microbiome, reduce gut inflammation, and ultimately impact brain inflammation via the gut-brain axis (Royes, 2020). Although these causal mechanisms remain contentious, the anti-inflammatory effects of exercise as a therapeutic strategy for AD are viable. Therefore, suppressing inflammatory responses may be a potential target for improving AD.

### 6.1 Exercise reduces inflammatory responses in microglia

In the current study of AD and other neurodegenerative diseases, exercise as a non-drug intervention has shown significant regulatory effects on microglia inflammation. Recent research suggests that exercise influences microglial activation states, promoting a shift toward profiles associated with anti-inflammatory and neuroprotective functions (de Almeida et al., 2022). This transformation is facilitated by complex signaling pathways, including the adiponectin/AdipoR1 axis. The adiponectin/AdipoR1 axis, crucial for interacting with the BBB, engages microglial AdipoR1 receptors. This interaction initiates signaling cascades that reduce the production of inflammatory cytokines by inhibiting NF-κB pathways and enhancing responses that protect neuronal integrity (Liu et al., 2024). Furthermore, the role of peroxisome proliferator-activated receptors (PPARs) in exercise-induced neuroprotection has been highlighted (Zhao et al., 2016). PPARs, especially PPAR-γ, are nuclear receptors that regulate genes involved in energy metabolism, lipid metabolism, and inflammation. Exercise-induced activation of PPAR-γ results in the enhancement of anti-inflammatory processes and the suppression of pro-inflammatory cytokines (Diniz et al., 2021), providing neuroprotection against the neuronal damage observed in neurodegenerative diseases like AD. The modulation of microglial activation by PPAR-γ agonists, such as pioglitazone and rosiglitazone, has been demonstrated to confer protective effects on neurons by leveraging the anti-inflammatory effects of exercise-induced PPAR-γ activation. Additionally, the neuroprotective role of exercise extends to enhancing levels of BDNF, which is crucial for maintaining and repairing neuronal health. Physical exercise elevates BDNF levels, thereby supporting synaptic plasticity and cognitive functions, and mitigating the adverse effects of aging and stress on neural circuits (Ben-Zeev et al., 2022). This interaction of exercise with molecular pathways that regulate BDNF expression underscores the potential of regular physical exercise to influence neuroplasticity and enhance cognitive resilience (Huang et al., 2023). These molecular insights reveal the potential of targeted exercise regimens as non-pharmacological interventions to modulate microglial functions and neuroinflammation, thereby improving cognitive and mental health. Continued exploration of these pathways not only promises to deepen our understanding of neuroimmune interactions but also paves the way for novel therapeutic strategies in neurodegeneration and psychiatry.

### 6.2 Exercise reduces inflammation in astrocytes

Similar to microglia, exercise also regulates inflammation in astrocytes, particularly through resistance training (Li et al., 2021). Kelty’s study have illuminated how resistance training and other forms of physical exercise intricately modulate astrocytic functions and neuroinflammation (Kelty et al., 2022). Resistance training, a specific form of physical exercise, has been shown to mitigate reactive astrocyte activation. This shift is crucial for mitigating the neuroinflammatory responses that contribute to cognitive decline and neuropathology. Mechanistically, this modulation prominently involves the upregulation of neuroprotective proteins such as BDNF and GDNF. These proteins are crucial for supporting neuronal survival and enhancing synaptic plasticity, thereby contributing to improved neurological functions (Fahimi et al., 2017; Piotrowicz et al., 2019; Belaya et al., 2020). Concurrently, the modulation involves suppression of inflammatory cytokines, achieved through the activation of intracellular signaling pathways. These pathways, which include calcium signaling and mitochondrial bioenergetics adaptations, are instrumental in reducing cellular stress and inflammation, thus protecting neural integrity. Moreover, exercise influences astrocytic function through epigenetic modifications that enhance their adaptive responses and resilience to stress. Recent findings indicate that exercise suppresses the production of neurotoxic proteins such as LCN2, involved in various neurodegenerative conditions (Jung et al., 2023), by modulating enzymes like α2-Na^+^/K^+^ ATPase. This enzyme plays a pivotal role in regulating astrocyte reactivity, chemokine production, and overall inflammatory milieu within the central nervous system. Furthermore, the neuroprotective effects of exercise extend beyond simple phenotype shifts in astrocytes. Exercise has been shown to induce systemic changes that affect astrocytic gene expression patterns related to plasticity and inflammatory response. These changes are believed to be mediated through complex interplays involving neurotrophic factors like BDNF, and through the suppression of pro-inflammatory pathways, notably via NF-κB signaling (Fahimi et al., 2017). Collectively, these studies underscore the potential of regular physical exercise as a non-pharmacological strategy to regulate astrocytic functions, reduce neuroinflammation, and protect against neurodegeneration. As research continues to delve into the molecular mechanisms by which exercise impacts astrocytes and other glial cells, it opens up promising avenues for integrating exercise into comprehensive treatment paradigms aimed at improving cognitive and mental health in populations at risk for or suffering from neurodegenerative disorders.

### 6.3 Exercise reduces inflammation in oligodendrocytes

Exercise also has a significant regulatory effect on inflammation in less synaptic cells. Some studies have found that physical exercise enhances the production of neurotrophic factors such as BDNF and Neurotrophin-3 (NT-3), which are critical for the development and maturation of OPCs (Pak et al., 2018). In AD, where neural degeneration and synaptic loss are prevalent, these factors promote the differentiation of OPCs into mature oligodendrocytes. This action is crucial as it leads to enhanced myelin repair and synthesis, improving neural transmission and potentially alleviating cognitive symptoms associated with AD. The anti-inflammatory effects of exercise are particularly beneficial in the AD context, as neuroinflammation is a hallmark of the disease’s progression. Exercise reduces the production of pro-inflammatory cytokines within the CNS while enhancing anti-inflammatory cytokines. This shift in cytokine profile protects oligodendrocytes from inflammatory damage, preserves myelin integrity, and supports overall CNS functionality. By suppressing NF-κB signaling—a key pathway that exacerbates inflammation—exercise helps in maintaining a healthier neuroinflammatory environment, which is crucial for slowing AD’s progression. Moreover, exercise improves mitochondrial function in oligodendrocytes, facilitated through the activation of PGC-1α (Kann, 2023). This improvement is vital because efficient mitochondrial function supports the energy-demanding process of myelin synthesis, essential for maintaining neuronal health and function in AD. Enhanced mitochondrial health in oligodendrocytes also combats oxidative stress, which is commonly observed in neurodegenerative diseases and can exacerbate AD pathology. Exercise also induces adaptive cellular stress responses that benefit oligodendrocytes. For example, the upregulation of heat shock proteins (HSPs) and other protective chaperones helps maintain protein homeostasis and protect oligodendrocytes from apoptosis (Richter-Landsberg and Bauer, 2004). This protection is particularly important in AD, where cellular stress and damage can accelerate disease progression. Collectively, these mechanisms underscore the therapeutic potential of regular physical exercise in influencing oligodendrocyte health, enhancing myelin integrity, and moderating neuroinflammation in AD. This supports the idea that tailored exercise regimens could be a strategic non-pharmacological intervention to mitigate the progression of AD, highlighting the importance of integrating Physical exercise into treatment and prevention strategies for those at risk or suffering from this neurodegenerative disorder. Through these multifaceted effects, exercise not only maintains CNS health but also offers a promising avenue for slowing the neurodegenerative processes characteristic of AD.

### 6.4 Exercise reduces damage to the blood-brain barrier in AD

Regular exercise has emerged as a significant non-pharmacological strategy that may mitigate the progression of AD by enhancing the integrity of the BBB. Exercise bolsters antioxidative defenses and endothelial function in the brain, which can improve the structural and functional aspects of the BBB (Liang et al., 2023). This is crucial, as the BBB’s integrity is pivotal in preventing neurotoxic substances from entering the brain, thereby slowing neurodegenerative processes (Liang et al., 2023). Moreover, exercise reduces systemic inflammation through mechanisms independent of weight loss, notably by decreasing the release of inflammatory cytokines such as TNF-α and IL-6 from skeletal muscles. This reduction in inflammation is beneficial for maintaining BBB integrity and has broader implications for neurovascular health, which is often compromised in AD. Another pathway through which exercise may protect the BBB is by modulating the metabolism of tryptophan along the kynurenine pathway. This adjustment leads to increased production of neuroprotective kynurenic acid and decreased production of neurotoxic metabolites that exacerbate BBB breakdown and neurodegenerative changes. Exercise also potentially influences genetic and molecular pathways associated with AD risk, such as those involving Apolipoprotein E ε4 (ApoEε4), a known promoter of BBB dysfunction (Montagne et al., 2020). By modifying the effects of such genetic risk factors, Physical exercise may offer protective benefits against the early onset and progression of AD, highlighting the multifaceted role of exercise in disease management and prevention. Incorporating regular physical exercise into daily routines could, therefore, be a key component of strategies aimed at delaying the onset or progression of AD through its multifarious effects on brain health and BBB integrity.

### 6.5 Exercise reduces inflammatory responses in peripheral circulation

Physical exercise not only modulates the inflammatory environment in the brain but also effectively regulates inflammation in the peripheral circulatory system, particularly in the gastrointestinal tract. Research indicates that regular exercise significantly impacts gastrointestinal health by modulating inflammation, enhancing the integrity of the gut barrier, and influencing the composition of the gut microbiota (Scheffer and Latini, 2020). These effects are pivotal for maintaining optimal gut function and preventing inflammatory diseases. Exercise improves the structural aspects of the gastrointestinal tract by promoting the thickness and strength of the intestinal wall, which can prevent the permeability that often leads to inflammation. In animal models of AD, where gut abnormalities mimic human gastrointestinal dysfunctions like increased intestinal permeability and inflammation, exercise has been shown to prevent the loss of wall thickness and mitigate inflammatory markers in the intestine, suggesting a protective structural effect. Moreover, the anti-inflammatory effects of exercise extend to the microbial inhabitants of the gut. Exercise induces shifts in the microbial community, increasing the diversity and abundance of beneficial bacteria such as those producing short-chain fatty acids (SCFAs) (Liu et al., 2021). SCFAs, particularly butyrate, are known to have potent anti-inflammatory properties and play a crucial role in maintaining mucosal integrity and promoting immune homeostasis. These fatty acids act as energy sources for epithelial cells in the colon, strengthen the gut barrier, and modulate inflammatory responses, thereby reducing the risk of inflammatory diseases. Exercise also has a systemic effect on inflammation (Xing et al., 2022). It reduces the levels of circulating inflammatory cytokines, such as TNF-α and interleukins, which are often elevated in chronic inflammatory states. By lowering these inflammatory mediators, exercise not only benefits the gut but also contributes to the overall anti-inflammatory status of the body. In addition to these biochemical impacts, physical exercise enhances gastrointestinal motility, which is beneficial for reducing symptoms of gastrointestinal disorders such as constipation. Improved motility helps in the regular evacuation of the bowels, which is crucial for maintaining a balanced microbiota and preventing the overgrowth of pathogenic bacteria that can trigger inflammatory responses. In summary, the beneficial effects of exercise on the gastrointestinal system are comprehensive, involving enhancements in physical structures, microbial composition, biochemical pathways, and overall gut function. These interconnected benefits underscore the potential of regular physical exercise as a non-pharmacological approach to managing and preventing gastrointestinal inflammation and related disorders.

## 7 Conclusion

This review synthesizes current insights into the impact of physical exercise on neuroinflammatory and neurodegenerative processes in AD. It examines how exercise modulates cellular functions within microglia, astrocytes, and oligodendrocytes, as well as its effects on the BBB and gut microbiota. Current literature suggests that physical exercise promotes anti-inflammatory and neuroprotective functions in microglia, moving beyond the simplistic binary of “pro-inflammatory M1” to “neuroprotective M2” phenotypes. Exercise also enhances the neuroprotection and repair capabilities of astrocytes, not merely through phenotype shifts from “A1” to “A2,” but by influencing gene expression and reducing inflammatory cytokine production, thereby improving synaptic support and overall brain health (de la Rosa et al., 2020).

Additionally, this review highlights the role of physical exercise in maintaining oligodendrocyte integrity, crucial for myelin sheath repair and neuronal function, with potential to slow AD progression. Research shows that exercise creates a supportive environment for the maturation of oligodendrocyte progenitor cells and boosts their myelinating abilities (Zou et al., 2023). It also confirms the beneficial impact of physical exercise on BBB integrity, citing studies that structured exercise regimens improve vascular function and decrease biomarkers of neuroinflammation in older adults (Malkiewicz et al., 2019).

The efficacy of exercise interventions depends significantly on their intensity and type. Moderate-intensity exercises like brisk walking or cycling are particularly effective, while more vigorous or inconsistent regimens may not provide similar neuroprotective benefits. This highlights the need for personalized exercise programs that consider the physical capabilities and disease progression stages of individuals with AD.

Challenges remain in the variability of exercise protocols and the scarcity of long-term studies on the sustained effects of exercise interventions on disease progression. The adaptability and effectiveness of these interventions across different stages of AD also warrant further exploration. Future research should prioritize longitudinal studies to refine exercise prescriptions and enhance their neuroprotective impact in AD. Additionally, exploring synergies between physical exercise, dietary factors, and pharmacological treatments could yield more comprehensive, multi-modal intervention strategies for AD.

## Data Availability

The original contributions presented in this study are included in this article/supplementary material, further inquiries can be directed to the corresponding authors.
